# The Role of Health Literacy in Adults' Dental Service Utilisation: A Scoping Review

**DOI:** 10.1111/cdoe.70037

**Published:** 2025-10-30

**Authors:** Xuanyun Lu, Sucharita Nanjappa, Thushani Indumani Devi Wijesiri, Peter Mossey, Siyang Yuan

**Affiliations:** ^1^ School of Dentistry University of Dundee Dundee UK

**Keywords:** communication, dental attendance, dental service, health literacy, oral health, service utilisation, utilisation

## Abstract

**Objectives:**

Health literacy refers to an individual's ability to understand health information, navigate healthcare systems, make informed decisions and adopt health‐promoting behaviours. The scoping review examined the available literature to explore adults' health literacy levels, health literacy measurements, and the role of health literacy in adults' dental service utilisation.

**Methods:**

The scoping review used Arksey and O'Malley's framework, refined by Joanna Briggs Institute methodology. The inclusion criteria were peer‐reviewed studies, published in English with adult participants in a dental setting. Four databases (MEDLINE via PubMed, Scopus, CINAHL and ASSIA) were searched for relevant studies from January 2000 to January 2025.

**Results:**

Nineteen studies met the inclusion criteria, with 12 out of 19 conducted in the US. Health literacy was assessed by 11 different measures. Although most studies reported relatively inadequate health literacy levels, inconsistent findings persist due to a lack of consensus on measurement. Dental service utilisation, primarily assessed by dental visits, dental information seeking, and dentist‐patient communication, demonstrated inconsistent associations with health literacy.

**Conclusions:**

While some studies suggested a positive association between health literacy and dental service utilisation, the mechanisms through which health literacy influenced dental service utilisation remained unclear and required further investigation.

## 1 Background

Health literacy is defined by the WHO (2021) as the knowledge and skills enabling individuals to access, understand, appraise and use health information and services to improve and maintain personal and community health [1]. It enables individuals to understand health information, advocate for their health needs, navigate and access healthcare systems, engage in informed decision‐making and foster health‐promoting behaviours [2, 3]. Despite its importance, a significant proportion of the global population has limited health literacy, which is often influenced by low general literacy [4]. For instance, in 2022, approximately 7.1 million adults in the UK read at or below the level of an average 9‐year‐old, thereby hindering their ability to process health information and advice [5]. Similarly, recent data from the U.S. showed over two‐thirds of adults demonstrated only ‘basic’ or ‘below basic’ health literacy [6]. These disparities in health literacy are particularly evident among disadvantaged groups of low socioeconomic status, who frequently encounter challenges such as language barriers, cultural differences and limited education [7].

Despite the well‐established association between low health literacy and poorer general health outcomes, such as reduced use of preventive care, poor medication adherence and higher hospitalisation rates [4], its impact on oral health and particularly its role in shaping dental service utilisation is underexplored. Dental service utilisation, which refers to the actual use of dental services, is distinct from access to care, which encompasses the opportunity to obtain services based on approachability, acceptability, availability, affordability and appropriateness [8]. While access enables care opportunities, utilisation reflects actual engagement with services. For example, high reliance on emergency care may indicate poor access to primary dental care [9]. Understanding this distinction is vital for addressing inequities, as disparities in utilisation often indicate structural access barriers rather than low patient demand.

Lower health literacy tends to reduce dental care utilisation with an adverse effect on oral health outcomes [10]. Health literacy facilitates the understanding of the importance of regular dental check‐ups, which promote disease prevention and early detection [11, 12] and reduce their reliance on emergency dental care [5]. Recent evidence showed only 43% of adults in England had visited an NHS dentist in the past 2 years [13], compared to 66% of U.S. adults within 1 year [14]. These discrepancies in dental service utilisation highlight the need to explore how underlying factors such as health literacy shape dental service utilisation patterns in different contexts.

While health literacy is widely recognised as a key factor influencing dental service utilisation, the literature suggests conflicting findings: some studies showed a positive association between health literacy and dental visits, whereas others reported no association [15, 16, 17]. This highlights the need for a more nuanced understanding of the role of health literacy in people's dental service utilisation. The aim of the scoping review was to explore the available research on the role of health literacy in adults' utilisation of dental health services with specific objectives to:

- determine adults' health literacy levels and its measurements,
- explore the role of health literacy in the utilisation of dental services.

## 2 Methods

A five‐step Arksey and O'Malley framework [18] refined by the Joanna Briggs Institute (JBI) [19] was applied, using the Preferred Reporting Items for Systematic Reviews and Meta Analyses extension for Scoping Reviews (PRISMA‐ScR) checklist [20] (Table S1). The steps of the Arksey and O'Malley framework [18] were as follows:

### 2.1 Stage 1: Identify the Research Questions

The research question was: what is the role of health literacy in adults' utilisation of dental health services?

### 2.2 Stage 2: Identifying Relevant Studies

The search strategy was applied to four databases including MEDLINE via PubMed, Scopus, CINAHL and the Applied Social Sciences Index & Abstracts (ASSIA). The MEDLINE via PubMed search strategy was developed using medical subject headings (MeSH) and text words related to health literacy and dental service utilisation to search for papers published from January 2000 to January 2025. The early 2000s marked a shift from general literacy to health literacy as a distinct research area with significant advancements in conceptual frameworks and standardised health literacy measures [21, 22]. The search terms are shown in supplementary documents (Table S1). This search strategy was adapted to the other three databases. After completing all the electronic database searches, the results were managed using the software EndNote X9 [23]. Reference lists of identified articles were manually searched to ensure that all relevant articles were identified.

### 2.3 Stage 3: Study Selection

The study selection followed inclusion and exclusion criteria based on the PCC (Population, Concept, Context) model (Table 1). Included studies were peer‐reviewed, published in English, with adults in a dental setting. After searching, duplicates were removed, and the remaining studies were transferred to RAYYAN software [24] and separately screened by two reviewers (XL and TIDW). Titles and abstracts were screened, with excluded articles removed. A third reviewer (SY) provided input when needed. Finally, full‐text reading was conducted for all the eligible articles.

**TABLE 1:** Inclusion and exclusion criteria.

Criteria type	Inclusion criteria	Exclusion criteria
Study design	All primary and secondary research including quantitative and qualitative research, all descriptive and analytical study designs	Opinion and position papers, editorials, newspaper and magazine articles
Population	Adults aged 18 years and above	Child and adolescent aged less than 18 years Adults with learning disabilities, cognitive developmental problems or mental disorder
Concept	The role of health literacy (including oral health literacy) on the utilisation of dental health services (including but not limited to seeking health information/service, access and use of dental health services, dental healthcare experience)	Studies that do not focus on the role of health literacy in adults' utilisation of dental health services
Context	Dental setting	Non‐dental settings
Language	English	Non‐English
Year of publication	Published between January 2000 and January 2025	Published year before 2000

### 2.4 Stage 4: Data Extraction

Relevant data were extracted and summarised under categories according to author, year of publication, study design and key findings.

### 2.5 Stage 5: Collating, Summarising and Reporting the Results

**FIGURE 1:**
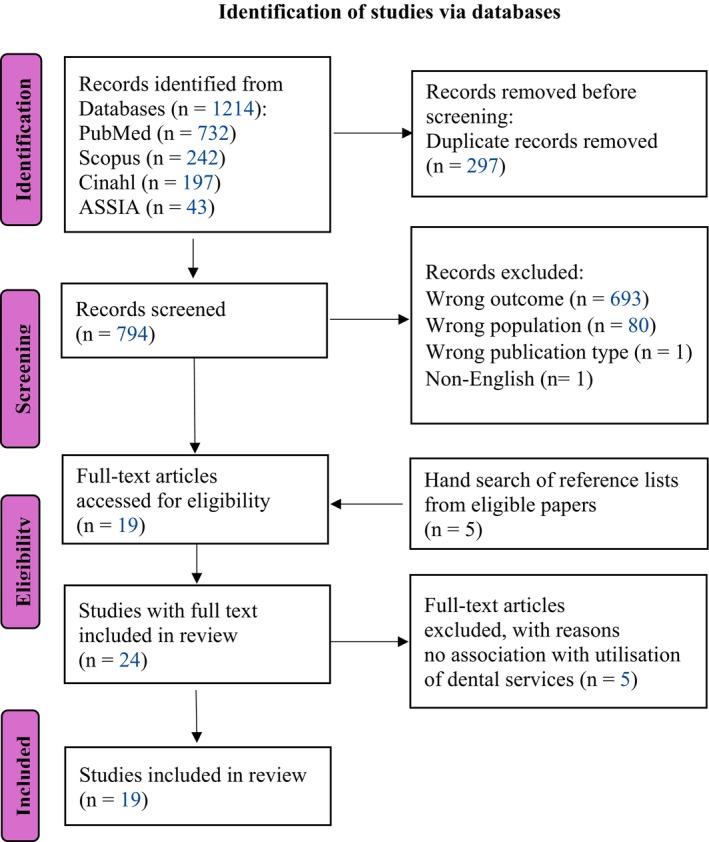
PRISMA‐ScR flowchart.

The results were summarised in a PRISMA‐ScR flowchart [20] (Figure 1). Data were extracted from all included studies, and a summary table of the results was synthesised as described in the Results section.

## 3 Results

Based on the eligibility criteria, a total of 19 articles were included after screening the abstracts and full texts of the articles (Figure 1).

### 3.1 Characteristics of the Included Studies

All the included studies were conducted in the past two decades in eight countries: the US (*n* = 12), Iran (*n* = 1), Australia (*n* = 1), Canada (*n* = 1), Egypt (*n* = 1), Brazil (*n* = 1), Indonesia (*n* = 1), India (*n* = 1), with 16 done in the last decade. Participants were adults aged 18 to 90 years with diverse demographics in terms of ethnicity, age, education and socioeconomic status. Fourteen studies targeted the general population, and the rest focused either on women [25] or refugees [26, 27, 28].

Fourteen out of 19 studies (*n* = 14) were cross‐sectional studies [16, 25, 26, 27, 28, 29, 30, 31, 32, 33, 34, 35, 36, 37], other study designs included two case–control studies [38, 39], a secondary data analysis [40], a cohort study [41] and a systematic review [17].

### 3.2 Health Literacy

Health literacy was assessed using various measures. Two studies used multiple health literacy measures [16, 25], while 16 used only one measure and one did not specify the measure [17]. Eleven measures were identified, with eight focused on oral health literacy, including the Rapid Estimate of Adult Literacy in Dentistry (REALD‐30) (*n* = 4) [16, 30, 39, 41], Comprehensive Measure of Oral Health Knowledge (CMOHK) (*n* = 3) [25, 32, 38], Health Literacy in Dentistry Scale (HeLD‐14) [29], Health Literacy (baseline survey) [26], Rapid Estimate of Adult Literacy in Medicine and Dentistry (REALM‐D) [40], Oral Health Literacy Adults' Questionnaire (OHLAQ) [27], Oral Health Literacy Instrument (OHLI) [28] and Indonesian Oral Health Literacy Questionnaire [36]. General health literacy was measured using the Test of Functional Health Literacy in Adults (Short‐TOFHLA) (*n* = 3) [16, 25, 30] and a dichotomised scale in one study [31]. Furthermore, one study employed the Basic English Skills Test (BEST Plus) for general literacy [16] (Table 2).

**TABLE 2:** Measurements of health literacy and dental utilisation types.

Categories	Measurements	Information	Score range	Low level	Medium level	High level
*Measurements of oral health literacy, health literacy and literacy*						
Oral health literacy measurements (*n* = 8)	Rapid Estimate of Adult Literacy in Dentistry (REALD‐30) [25, 28, 29, 32, 37, 39]	This is a basic word recognition test with a score range from 0 to 30	0–30	≤ 21	22–25	≥ 26
	Comprehensive Measure of Oral Health Knowledge (CMOHK) [24, 31, 33, 36]	This is a 23‐item questionnaire assessing knowledge on the prevention and management of dental caries, periodontal disease and oral cancer. It is a validated instrument	0–23	0–11	12–14	15–23
				0–14		15–23
	Health Literacy in Dentistry scale (HeLD‐14) [16]	This is a short‐form, 14‐item version with a score ranged from 0 to 56. Its questions are designed to assess oral health literacy in seven domains: communication, understanding, utilisation, access, receptivity, support and financial resources	0–56	> 19		≤ 19
	Health literacy (baseline survey) [13]	It includes three validated questions assessing people's understanding of recognising words and reading, understanding of speaking, and their abilities of applying. The questions refer to how often they have problems understanding the written health related materials, understanding the providers speaking and filling out medical forms	0–3	Not stated	Not stated	Not stated
	Rapid Estimate of Adult Literacy in Medicine and Dentistry (REALM‐D) List 3 [38]	Similar as REALD‐30, this is a validated and reliable screening instrument for word recognition, created by adding dental terms to the existing 66‐item Rapid Assessment of Adult Medical Literacy (REALM). It is structured into three lists ranging from one‐syllable words in List 1 to the most difficult words in List 3	0–28	≤ 21.5		≥ 21.5
	Validated OHL adults' questionnaire (OHLAQ) [26, 35]	This is a 17‐items questionnaire divided into four sections: reading comprehension, numeracy, listening and decision‐making to measure the participants' understanding abilities, communication skills and applying skills	0–17	(Inadequate) 0–9	(Marginal) 10–11	(Adequate) 12–17
	Oral Health Literacy Instrument (OHLI) [27]	It measures reading comprehension and numeracy skills, scored on a scale of 0–100	0–100	(Inadequate) ≤ 59	(Marginal) 60–74	(Adequate) ≥ 75
	Indonesian Oral Health Literacy Questionnaire [34]	The questionnaire consisted of seven question items divided into five domains: communication, receptivity, understanding, utilisation and support	0–28	0–9	10–18	19–28
Health literacy measurements (*n* = 2)	Test of Functional Health Literacy in Adults (Short‐TOFHLA) [24, 28, 29]	This is a 36‐item reading comprehension test using a set of sentences from a medical scenario with keywords missing	0–36	Inadequate: 0–22		Marginal to adequate: 23–36
	A dichotomised scale [30]	It includes three indicators measuring how difficult it is to: Obtaining advice or information about health Understanding information from health professionals Understanding written health information	3–12	6–12		3–5
Literacy measurements (*n* = 1)	Basic English Skills Test (BEST Plus) [28]	It measures the spoken English proficiency of participants and their behavioural risk	Not stated	Not stated	Not stated	Not stated

	Utilisation types	Information	Specific outcomes		
*Measurements of dental utilisation types*					
Utilisation of dental health service (*n* = 4)	Dental visit (*n* = 18) [13, 16, 24, 25, 26, 27, 28, 29, 30, 31, 32, 33, 34, 35, 36, 37, 38, 40]	It is measured by whether a patient went to the dentist, dental hygienist, or other dental care provider in the past time span designed by the investigator. And times of visiting dentist	Reason for visiting [13, 28, 29, 31, 40]	Frequency of visiting [16, 24, 26, 27, 30, 31, 37, 40]	Missed dental appointments [36, 38]
	Dental information seeking (*n* = 3) [27, 36, 38]	It is measured by frequency of seeking dental information, the sources of dental information and the number of health information sources used	Frequency of seeking dental information [36]	Sources of dental information [27, 36]	Number of sources used [38]

	Dentist‐patient communication (*n* = 3) [13, 27, 36]	It is measured by: The patient's self‐report communication skills through answering four questions: ‘Whether they can ask their dentist questions; Whether they can understand the dentist; Whether they are able to use dental health information to make decisions; And whether they are able to find another dentist for second opinions’ [36] Dental treatment decision‐making by answering whether they participated in the process of decision‐making [27] Patient's perceived interaction with dentist by answering 4 questions: ‘When you visit a dentist, how often does the dentist explain things in a way that is easy to understand? How often does the dentist listen carefully to you? How often does the dentist treat you with courtesy and respect? And how often does the dentist spend enough time with you?’ [13]	Patient's self‐report communication skills [36]	Participation of treatment decision‐making [27]	Perceived interaction with dentist [13]
Dental neglect (*n* = 1) [39]	It is measured by the patients' self‐report through answering for 6 questions about their dental behaviours, which described their avoidance of dental care. The six items are ‘I keep my dental care at home’; ‘I receive the dental care I should’; ‘I need dental care, but I put it off’; ‘I brush as well as I should’; ‘I control snacking between meals as well as I should’; and ‘I consider my dental health to be important’	Avoidance of dental care [39]		

Twelve studies described health literacy levels using mean scores from various instruments [16, 25, 28, 29, 30, 32, 38, 39, 40, 41] which were either categorised into three levels (low, medium, high) or two levels (low and high). There was no consensus on the cut‐off score for identifying the adequacy of health literacy levels due to their different focuses on various dimensions and targeted populations. Among these 12 studies, seven reported inadequate health literacy levels [16, 25, 29, 30, 32, 39, 41], while five reported medium or adequate levels [28, 38, 40]. The remaining seven articles did not report mean scores of health literacy [17, 26, 27, 31]. (Table 3). Additionally, disparities in health literacy were found among participants based on demographics such as ethnicity, age, gender, education, language competence and socioeconomic status [16, 29, 30, 32].

**TABLE 3:** Summary of key findings.

Author/year	Levels of health literacy	Utilisation types	Key findings
Baskaradoss (2016) [38]	The mean CMOHK score indicated high Oral Health Literacy (OHL)	1. Frequency of seeking dental information and the sources of dental information 2. Whether patients missed dental appointments 3. Patients' communication skills	There was no significant difference for seeking dental information between the patients who had at least one missed appointment within 12 months of the study and patients who had kept all scheduled appointments during the same period. Poor OHL was found to be independently associated with missed dental appointments. There was no difference between patients with various health literacy levels on their communication skills.
Geltman, et al. (2014) [28]	74% of the participants had low health literacy (HL) measured by STOFHLA 73% of the participants had low REALD score 56% of the participants had low BEST+ score	1. Whether participants had a dental visit in the past 12 months 2. The purpose of dental visits 3. Whether participants had a preventive visit in the past 12 months	When acculturation was controlled for, no significant difference was found in using preventive care between high and poor HL groups.
Guo, et al. (2014) [13]	No indication of OHL level of the participants measured by Health literacy (baseline survey) instrument	1. Dental care patterns: (regular/problem‐oriented) 2. Patient's perceived interaction with dentist	Higher levels of HL were associated with better dentist‐patient communication, which in turn corresponded with regular attendance of dental care.
Henderson, et al. (2018) [16]	68.4% of the participants had low HeLD score	Whether a patient went to the dentist, dental hygienist, or other dental care provider in the past 12 months	Patients with low OHL were 39% less likely to have visited the dentist in the past 12 months, compared to patients with high OHL.
Holtzman, et al. (2014) [38]	The mean REALMD score indicated high OHL	1. Dental information seeking measured by the number of health information sources used 2. Dental visit measured by whether patients attended or missed dental appointments	Seeking health information through fewer rather than multiple sources, was reported as the strongest predictor of missing an appointment. Low OHL scores emerged as the second strongest predictor of missing appointments.
Jones, et al. (2007) [37]	28.7% sample's REALD‐30 score indicated low OHL	Dental visit in the last year	Dental visit in the last year was not associated with health literacy.
Kino and Kawachi (2020) [30]	No indication of OHL level of the participants measured by a dichotomised scale of three indicators for health literacy information seeking and comprehension	Dental visit in the past year	There was a moderating effect of health literacy on dental visits among insured individuals: those with higher HL level tended to have more dental visit compared to those with low HL. Conversely, among uninsured people, lower health literacy was associated with more frequent dental visit.
Nguyen, et al. (2022) [31]	The mean OHL score measured by CMOHK indicated low OHL	Dental care use in the past year including frequency of dental visits and reason for dental visits	When acculturation was measured including language proficiency, participants with both high acculturation and high OHL were significantly more likely to have dental visit compared to those with both low acculturation and low OHL.
Sistani, et al. (2017) [26]	No indication of OHL level of the participants measured by validated OHL adults' questionnaire (OHLAQ)	Dental visit during the previous 6 months	OHL significantly improved oral health behaviour (more daily tooth brushing and less consumption of sugary snacks or beverages) and the dental visit during the previous 6 months.
Firmino, et al. (2018) [40]	Systematic review without clear indication of the HL level	Frequency of dental visit and reason for the last dental visit	No association was found between OHL and the frequency of dental visit or the reasons for the last dental visit.
Parker and Jamieson (2010) [29]	The mean REALD‐30 score was 15 indicating low OHL	Problem‐based dental attendance	OHL was associated with problem‐based dental attendance. Patients with low OHL scores usually visited a dentist because of a dental problem and vice versa.
Macek, et al. (2017) [24]	18% of participants exhibited low conceptual knowledge measured by CMOHK	Dental visit	There was no significant association found between conceptual knowledge (a component of OHL) and dental visit or dental cleaning within 1 year.
Lee, et al. (2012) [39]	The mean REALD‐30 score was 15.8 indicating low OHL	The patients' self‐report through answering for 6 questions about their avoidance of dental care	OHL showed no association with patients' avoidance of dental care. However, when OHL scores combined with self‐efficacy, significantly association was found with dental neglect.
Calvasina, et al. (2016) [27]	The mean OHL score was 83.4, which is adequate OHL measured by OHLI	1. Dental visit measured by the times of dental visit within 1 year 2. Dental information seeking 3. Dental treatment decision making (communication)	Limited OHL was associated with lower participation in the oral healthcare and with barriers to using dental services. Specifically, inadequate/marginal OHL was associated with not visiting a dentist in the preceding year, not having a dentist as the primary source of dental information, not participating in shared dental treatment decision making
Badran, et al. (2023) [32]	The mean HL score was 25.9 measured by Arabic Rapid Estimate of Adult Literacy (ARELAD‐30), which is medium level of OHL	Time since last dental visit	Higher A‐REALD scores indicating good dental literacy being associated with less time since last dental visit.
Adunola, et al. (2023) [33]	Did not state the mean score of OHL and participants' levels of HL	Urgent care utilisation for a dental problem	CMOHK scores were not significantly associated with visits to the ER and/or urgent care.
de Araujo, et al. (2024) [25]	70% of participants showed high OHL meased by Brazil REALD‐30	Frequency of use of dental services	Higher REALD‐30 score was associated with less frequency use of dental service.
Oktadewi, et al. (2024) [34]	Did not state the mean score of OHL Only 1% of respondents had a poor OHL level meased by Indonesian Oral Health Literacy Questionnaire	Dentist visit frequency, and the first action taken when experiencing toothache	There was no correlation between OHL and dentist visit frequency, and the first action taken when experiencing toothache.
Chalapathi, et al. (2025) [35]	Did not state the mean score of OHL measured by Oral Health Literacy Adult Questionnaire (OHLAQ)	Demand for the replacement of missing teeth	OHL scores were directly related to needs for the replacement of missing teeth.

Abbreviations: BEST, Basic English Skills Test; CMOHK, Comprehensive Measure of Oral Health Knowledge; HeLD‐14, Health Literacy in Dentistry scale; HL, health literacy; OHL, oral health literacy; OHLAQ, validated oral health literacy adults' questionnaire; OHLI, Oral Health Literacy Instrument; REALD‐30, Rapid Estimate of Adult Literacy in Dentistry; REALM‐D, Rapid Estimate of Adult Literacy in Medicine and Dentistry; TOFHLA, Test of Functional Health Literacy in Adults.

### 3.3 Dental Service Utilisation

Eighteen studies used dental visit [16, 17, 25, 26, 27, 28, 29, 30, 31, 32, 33, 34, 35, 36, 37, 38, 39, 40] including frequency and number of visits, to measure dental service utilisation. In addition, dental information seeking (*n* = 3) [26, 28, 38] was assessed as a measure for dental service utilisation by frequency, sources and the amount of information sought. Dentist–patient communication (*n* = 3) [28, 38, 40] was identified as another way to measure service utilisation. Interestingly, dental neglect (*n* = 1), measured by self‐reported avoidance of dental care, was used in one study [41]. Ten studies used one measure for dental utilisation [16, 17, 25, 27, 29, 30, 31, 32, 39, 41], whereas four explored multiple measures [26, 28, 38, 40] (Table 2).

### 3.4 The Role of Health Literacy in Utilisation of Dental Services

Among 18 articles that investigated the role of health literacy in dental visits, six revealed a positive association with dental visit frequency [16, 27, 28, 29, 30, 31] but one reported a negative association [35]. One study reported a negative association with time since last dental visit [33]. One reported a positive association with the need for the replacement of missing teeth [37]. One article presented a complex association, indicating a positive association between health literacy and regular dental attendance and a negative association with problem‐based dental visits [26]. Six articles found no association between health literacy and dental visits [17, 25, 32, 34, 36, 39] (Table 3).

In terms of dental information seeking as the measure for service utilisation (*n* = 3), two articles found a significant positive relationship that individuals with adequate health literacy were more likely to use reliable information sources, such as relying on their dentists as the primary source [28, 40]. One study, however, reported no association [38].

Among the three articles exploring the effect of health literacy on dentist–patient communication, two suggested that higher health literacy levels led to better perceived communication [26] and increased participation in decision‐making [28]. Nevertheless, one study found no impact on patients' communication skills [38].

No significant association was found between health literacy and dental neglect [41]. However, when combining health literacy scores with self‐efficacy, a significant negative association with dental neglect emerged.

## 4 Discussion

Health literacy plays an active role in managing oral diseases, which are largely preventable and account for a significant proportion of global noncommunicable diseases [12]. This review found no consensus in terms of health literacy measurement used in dentistry. The health literacy level of adults remained inconclusive, with half reporting inadequate health literacy and the rest either showing adequacy or no indication of mean score. The role of health literacy in adults' dental services utilisation remained unclear due to inconsistencies in its measurement and study design. Despite 11 articles indicating a positive association with dental service utilisation, others found weak or non‐significant associations, making it difficult to draw definitive conclusions.

Significant challenges were encountered when collating the scoping review results due to a lack of consensus on health literacy measurement. This review identified 11 different instruments, each focusing on a different aspect of health literacy. For instance, REALD‐30 emphasised word recognition, whereas Short‐TOFHLA focused on functional health literacy, and CMOHK targeted conceptual knowledge. This lack of consensus complicates the reliable assessment of health literacy and has received considerable attention from researchers. A widely accepted, comprehensive measurement covering all health literacy dimensions is anticipated in the future, which would enhance understanding and facilitate the development of effective intervention strategies [42].

Seven articles reported inadequate health literacy levels among participants, which was consistent with previous studies highlighting limited health literacy as a global issue [12, 26]. The WHO has highlighted the urgency of improving health literacy as one of their seven health promotion strategies, as people with limited health literacy face challenges with understanding medical instructions, accessing appropriate care, and managing chronic conditions [43], which contribute to adverse health outcomes, increased healthcare costs, and disparities in healthcare access and utilisation [44].

The association between health literacy and dental visits is complex. Dental visits can be seen in two patterns, that is, preventive and problem‐based dental attendance, which showed different relationships with health literacy [35]. People with higher health literacy tended to better appreciate the value of regular dental examinations and were more likely to visit the dentist regularly [16, 28, 30]. Conversely, more problem‐based or irregular visits were observed in those with lower health literacy [40], as they struggled to access and understand health‐related information and often underestimated the importance of routine dental care in maintaining oral health.

Interestingly, six studies reported no association when dental service utilisation was measured by frequency of dental visits, with mixed reasons, in the past 6 or 12 months. This might indicate that simply using the number of dental visits to describe dental service utilisation may not truly reflect dental service utilisation. More visits do not necessarily imply optimal use of dental services, as individuals have varied purposes, needs and motivations [45, 46, 47]. For example, people might visit the dentist several times within a year for emergency care. Moreover, the relationship between health literacy and dental visits might be influenced by other factors such as socio‐demographics, healthcare systems and cultural contexts [45, 46, 47]. Future research is needed to better understand this relationship between health literacy and dental attendance.

Findings showed that individuals with sufficient health literacy were more likely to seek reliable health‐related information from sources such as dentists to make informed decisions about their health [28, 40, 48]. Yet, those with limited health literacy often relied on families, friends, leaders and religious institutions for health information instead of health professionals or reputed websites [48]. Therefore, developing health literacy is essential to empower people to obtain reliable dental information for themselves.

This review identified positive impacts of health literacy on dentist‐patient communication, which in turn improved patients' oral health outcomes [26, 28]. This was in line with evidence suggesting that dentist‐patient communication mediated the impact of health literacy on oral health outcomes [49]. Health literacy could facilitate effective communication by empowering patients to process health information, ask relevant questions and actively participate in health decision‐making [49]. This fostered a collaborative partnership between patients and dentists that promoted shared decision‐making, adherence to treatment plans and good health outcomes [49].

Communication is a key component of several theoretical frameworks for health literacy in healthcare. For instance, the transactional model of communication suggested that effective communication empowered patients to engage in preventive activities and regular check‐ups, thus enhancing overall health [50]. Moreover, other frameworks [51, 52] identified patient–doctor communication as a crucial mediator between health literacy and health outcomes, which was in line with the pathway found in dentistry [26].

### 4.1 Study Limitations and Research Gaps

This scoping review has several limitations. First, all included studies were published in English, which may have resulted in the exclusion of relevant articles published in other languages. In addition, only two studies were conducted in low‐ and middle‐income countries where health literacy is a more pressing issue, as indicated by the WHO [53]. Third, over half the studies relied on self‐report questionnaires and mostly used cross‐sectional design, which may generate reporting biases and not accurately reflect health literacy levels or dental service utilisation over time [54, 55]. Moreover, selection bias might exist, as participants could have higher baseline literacy levels due to their ability to understand and complete the surveys [56]. Finally, a systematic review was included in this study, which resulted in four articles being double‐counted. However, the aim and objectives of the systematic review were different from those of this scoping review.

This review identified gaps in research evidence. For instance, while disadvantaged groups often have poorer health literacy and reduced access to dental care [4, 53, 57], most included studies concentrated on the general population. Moreover, unlike in medical literature [4, 51], there is a paucity in dental research to explicitly explore the association between health literacy and dental service utilisation, resulting in inconsistent findings.

## 5 Conclusion

Seven included articles reported inadequate levels of health literacy in adults. The relationship between health literacy and adults' dental service utilisation remained unclear. While 11 studies suggested a positive association, the pathways through which health literacy influenced this behaviour were not well understood. It remained unclear how health literacy—such as understanding oral health information, navigating healthcare systems, or making informed decisions—impacted the use of dental services. Further research is needed to explore the underlying factors, such as living arrangement and income, and potential barriers like language competence and education level that may mediate or moderate this relationship.

## Conflicts of Interest

The authors declare no conflicts of interest.

## Supporting information


**Table S1:** Preferred Reporting Items for Systematic reviews and Meta‐Analyses extension for Scoping Reviews (PRISMA‐ScR) checklist.
**Table S2:** Search terms.

## Data Availability

Data will be available from the corresponding author upon reasonable request and subject to ethical approval.
